# Gas Leak in the Port: Portal Venous Gas Associated With Congenital Cytomegalovirus Colitis

**DOI:** 10.1097/PG9.0000000000000267

**Published:** 2022-10-25

**Authors:** Kentaro Tominaga, David Haslam, Akihiro Asai

**Affiliations:** From the * Division of Gastroenterology, Hepatology, and Nutrition, Cincinnati Children’s Hospital Medical Center, Cincinnati, OH; † Cincinnati Children’s Hospital Medical Center, Division of Infectious Diseases, Cincinnati, OH; ‡ Department of Pediatrics, University of Cincinnati College of Medicine, Cincinnati, OH.

A full-term 10-week-old male presented with persistent hematochezia. Physical examination revealed hepatomegaly. Growth was normal, without episodes of hypoglycemia nor signs of cardiac anomaly. Laboratory evaluation showed slight elevation of alanine transaminase (ALT) and aspartate transaminase (AST) with normal direct bilirubin and gamma-glutamyl transferase. Cytomegalovirus (CMV) was detected in urine and stools by PCR. The abdominal ultrasound showed echogenic particles flowing through portal veins, demonstrating portal venous gas (Video 1). The abdominal computed tomography was normal with no features of intestinal pneumatosis. Flexible sigmoidoscopy revealed multiple oozing ulcerations in the rectosigmoid colon (Fig. 1); histology showed nonspecific inflammatory cell infiltration without mucosal features of chronic ischemia. The biopsy samples were positive for CMV on PCR. He was treated with long-term oral ganciclovir for congenital CMV colitis, and hematochezia and portal venous gas resolved in 2 weeks. He is currently well at 18 months old.

**Figure:**
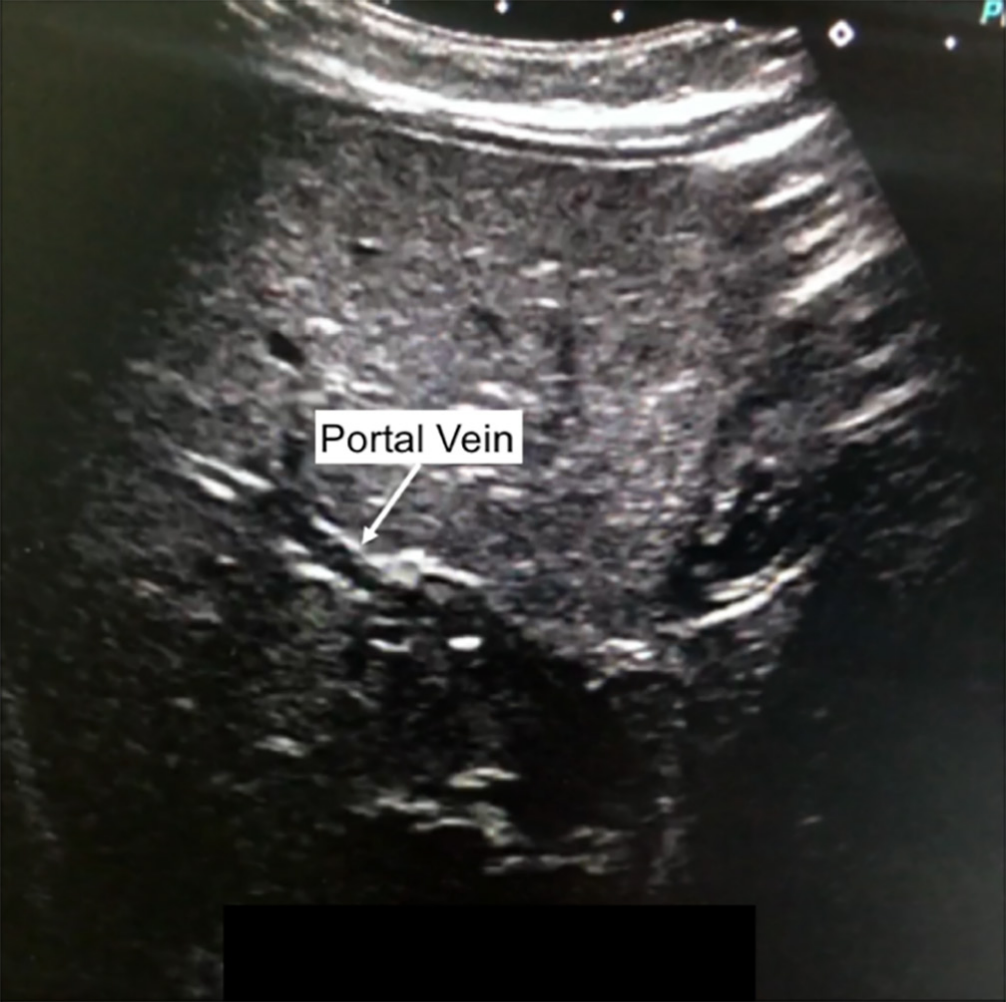
VIDEO 1. A Video clip of liver ultrasound images. Transverse imaging of the liver captured air bubbles in the portal vein.

**FIGURE 1.**
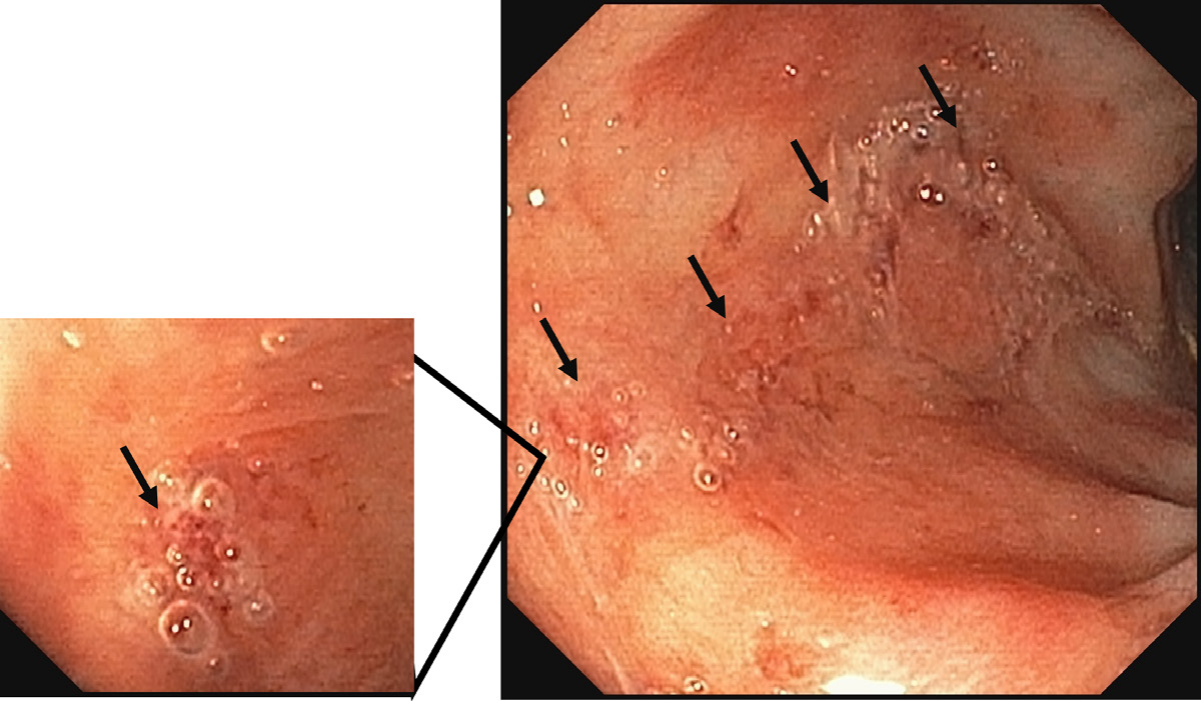
A photo capture image of the sigmoidoscopy. The black arrows indicate ulcers, which were covered with air bubble-containing secretion. The ulcers did not actively produce air bubbles after being flushed out with water.

Portal gas in infants is rare and often reflects severe damage to the intestinal mucosa. The most common cause of portal venous gas in children is necrotizing enterocolitis (NEC), with intestinal pneumatosis in premature newborns (1). Severe CMV colitis in immunocompromised adults can cause pneumatosis and portal venous gas. However, congenital CMV colitis has not been associated with portal venous gas (2,3). In a previous report, congenital CMV enterocolitis was diagnosed in 24.7% of cases with invasive congenital CMV infection among presumably immunocompetent newborns (4). Food protein-induced enterocolitis syndrome (FPIES) is an important differential diagnosis, because it can cause portal venous gas similar to NEC (5,6). Because of its higher prevalence, allergic proctocolitis can occur in conjunction with congenital CMV infection (7).

Our case lacked typical clinical features of NEC and FPIES. He was ultimately diagnosed with CMV colitis by PCR of colonic biopsy. This case highlights the importance of screening for congenital CMV colitis in infants with portal venous gas.

## ACKNOWLEDGMENTS

The corresponding author, Akihiro Asai, confirms that verbal informed consent was obtained from the patient for the publication of their information and imaging.
